# The search for a hippocampal engram

**DOI:** 10.1098/rstb.2013.0161

**Published:** 2014-01-05

**Authors:** Mark Mayford

**Affiliations:** Department of Molecular and Cellular Neuroscience, The Scripps Research Institute, La Jolla, CA, USA

**Keywords:** memory, hippocampus, circuits, genetics, calcium imaging, neural representation

## Abstract

Understanding the molecular and cellular changes that underlie memory, the engram, requires the identification, isolation and manipulation of the neurons involved. This presents a major difficulty for complex forms of memory, for example hippocampus-dependent declarative memory, where the participating neurons are likely to be sparse, anatomically distributed and unique to each individual brain and learning event. In this paper, I discuss several new approaches to this problem. *In vivo* calcium imaging techniques provide a means of assessing the activity patterns of large numbers of neurons over long periods of time with precise anatomical identification. This provides important insight into how the brain represents complex information and how this is altered with learning. The development of techniques for the genetic modification of neural ensembles based on their natural, sensory-evoked, activity along with optogenetics allows direct tests of the coding function of these ensembles. These approaches provide a new methodological framework in which to examine the mechanisms of complex forms of learning at the level of the neurons involved in a specific memory.

## 1. Introduction

It has been known for over 60 years that medial temporal lobe (MTL) structures are necessary for declarative or explicit forms of memory [1]. This memory for people, places or events is what the average person envisions when referring to memory and is a critical component of what makes us who we are as individuals. What is particularly surprising about the presentation of patients with MTL damage is the presence of severe memory deficits in conjunction with the sparing of other sensory and cognitive functions. For example, in patient HM, who underwent a bilateral resection of MTL structures, IQ measures increased slightly following surgery while his ability to form new declarative memories was profoundly impaired [2]. This suggests that MTL structures, in particular the hippocampus and surrounding entorhinal and perirhinal cortices, play a specialized role in this form of memory independent of other sensory and cognitive functions. Extensive study of the MTL in memory has revealed a number of clues to how it may function, however there are a number of technical and conceptual issues that hamper a more definitive understanding of this memory system.

To take a typical example of a declarative memory, let us say your mother phones you and informs you that she just bought a red sports car. The next time the topic of cars or your mother comes up there is a good chance that you will mention this bit of information. What would it mean to ‘understand’ the mechanisms of this specific memory? To begin the search, we must consider that you are not born with a genetically determined neural representation of either your mother or red sports cars, so essentially you are forming a new memory linking these two older memories. Thus, the first problem we encounter is that the component stimuli in a declarative task are often themselves complex and as represented in the brain are most probably sparsely and broadly encoded, moulded by past experience-dependent plasticity and unique to each individual at the level of the precise neurons involved. The problem is further compounded if we consider the engram or memory trace, defined as the cellular and molecular change in the brain that occurs with learning and alters the subsequent processing of specific external cues to produce memory recall. Which of the many neurons that respond to the two component stimuli are likely to be molecularly altered to provide this link that is at the heart of memory?

It is important to first clearly distinguish between the concept of a neural representation and a memory trace or engram. The term neural representation is most commonly used by physiologists studying sensory processing and refers to the information carried in a neuron's firing pattern in relation to the external world [3]. This is generally characterized by measuring the firing evoked by manipulation of external sensory cues. For example, in the primary visual cortex, neurons respond to basic structures in a visual stimulus, such as the orientation and direction of motion, and those neurons could therefore be said to represent these features of the stimulus [4]. The linkage of environmental stimulus features to neuronal firing has been described throughout the brain with perhaps the most striking examples coming from recordings of human hippocampus. In one study, recordings from electrodes implanted in the MTL of patients awaiting epilepsy surgery found neurons that responded to photos of a specific individual actress as well as to the written name of that actress [5]. The ability of these neurons to respond to both photos and textual stimuli of a specific individual suggest that they represent the concept of that individual rather than some higher order visual feature. However, as striking as these neural responses are, they are still just sensory-evoked correlations in firing. The demonstration that these neurons actually ‘represent’ the actress in question would require the alteration of perception with the direct manipulation of the activity of those specific neurons.

To return to the declarative memory example discussed above, suppose that we found a set of neurons that responded to photos of your mother, and moreover we demonstrated that we could artificially stimulate that specific set of neurons and the thought or perception of your mother popped into your head. In addition, after the phone call, the same set of neurons fired in response to photos of a red sports car, whereas previously they did not; would this result indicate that we had found the engram for this memory? No, we would have simply found a neural representation of your mother and we would have found that after learning this neural representation could be recruited by a new set of stimuli (Jaguars), but we would not have found the sites of plasticity that allowed the various external representations of Jaguars (photos, text, etc.) to be processed so as to now evoke the firing of the neural representation of your mother. While each of the two neural representations in this example have themselves been moulded by previous learning, we do not know the sites of plasticity that produced them, and so should not formally consider them engrams. Moreover, while these complex representations have now been linked in a new declarative memory, the responsive neurons that we record are not necessarily the site of the plasticity that underlies this association. This may occur in a portion of the neurons in each representation or at some upstream site of processing. A final question is: what is the difference between a perceptual representation and a memory? Clearly, memory recall is not experienced in the same way as actual sensory perception of the recalled event. How is this dissociation achieved? Are there completely separate representations for memory and sensory perception or are there specific components of the sensory representation that are recruited to give recognition without perception?

Given the constraints outlined above, what experimental criteria could be used to definitively identify an engram for a declarative memory? I suggest four requirements for the identification of a declarative memory engram, which parallel a set of criteria outlined by Martin *et al*. [6] for testing the hypothesis that NMDA-dependent long-term potentiation (LTP) is a cellular mechanism for learning. They are: (i) identify a learning-induced molecular and corresponding functional cellular change in a specific subset of neurons. (ii) Block the identified molecular/cellular change and prevent memory formation. (iii) Induce the identified molecular/cellular change in the identified subset of neurons or synapses and produce a memory independent of behavioural training. (iv) Determine how the learning-induced cellular changes function within the circuit to produce recognition (e.g. recruit specific neural representations) and alter behavioural output.

The hurdles to meeting these criteria are substantial. First, we lack an understanding of basic mechanisms of neural processing and how the complex representations intrinsic to declarative memory are produced. However, the most difficult issue is identifying, isolating and ultimately manipulating sparse populations of neurons that are anatomically distributed and unique to each individual brain. In this article, I outline some of the outstanding questions and limitations to understanding the mechanisms of declarative memory. I discuss some of the new tools being developed in animal models to address these problems and describe a framework for addressing the circuit structure and plastic changes associated with this form of memory.

## 2. Mapping neural representations in activity patterns

A central problem to beginning a dissection of declarative memory mechanisms is the complexity of the component stimuli. In order to begin to understand how this type of memory for people, places and events is formed, it would be helpful to have a clear description of how and where the component stimuli are represented in the brain at the level of specific neural ensembles. Rodent models of hippocampal-dependent memory have focused on place learning. The most commonly used tasks, the water maze, radial arm maze or T-maze, require the animal to use distal cues to navigate to a specific goal location in the environment. Hippocampal lesions impair spatial learning in these tasks while sparing performance on analogous cued versions [7,8]. Another place learning task that is sensitive to hippocampal lesion is contextual fear conditioning, which simply requires recognition of place rather than navigation to a particular location [9]. In this task, the animal receives foot-shocks in a conditioning chamber with multi-modal sensory cues (visual, olfactory and tactile) leading to a fear memory for the shock box (context) relative to similar chambers containing a distinct constellation of sensory cues.

How is this information for place or context represented in the brain? Electrophysiological recording of hippocampal activity in freely behaving rats found that one of the most striking features of hippocampal principal neurons is spatially specific firing [10]. When animals are allowed to freely move in an open field or on more restrictive tracks, individual hippocampal neurons are active only when the animal passes through a limited region of the environment, suggesting that they encode a map of the animal's spatial location [11]. These place cells have been the focus of a great deal of study over the past 40 years and they not only display a number of features that could contribute to spatial memory but also suggest a more general role in other forms of declarative memory. First, the place cell ensemble recruited is specific to the environment the animal is exploring but this specificity can take some time to develop [12]. This study examined place cells in animals exploring either a circular or square arena that differed only by shape and found that initially there was strong overlap in place cell ensembles in both environments that over repeated trials developed into distinct patterns for each arena. Moreover, once the arena-specific firing developed it remained stable for up to one month, suggesting a learned representation of the two environments in the hippocampus.

Hippocampal neurons are not responsive only to the animal's location in the environment. When running on linear tracks, hippocampal neurons show place fields that are sensitive to the direction in which the animal is moving [13]. In T-maze-type learning tasks where the animal is running on a track but then required to make a directional choice to receive a reward, neurons have been found that respond differentially during approach to the choice point, suggesting prospective coding of a navigational decision [14,15]. While spatial codes are prominent in the rodent hippocampus, in humans the hippocampus is critical for coding a much greater variety of information. Studying rats, Wood *et al*. [16] used a delayed non-matching to odour task, in which a food reward was associated with odour cues that were distinct from the previous trial (non-match), performed at random locations in an open field to test the behaviour of hippocampal neurons in a task that did not require the use of spatial information. In this test, they found that only 11% of cells had a purely location-specific firing correlate while 40% of cells had purely non-spatial firing. Of the non-spatial cells, there were neurons that responded to specific odours or to the match/non-match of the odour presented in a given trial. This demonstrates that even in the rodent, the hippocampus codes for non-spatial memory task-relevant information and depending on task demands this can comprise the predominant firing mode.

While current technology allows the recording of tens of neurons simultaneously with excellent temporal resolution of spike activity, the approach suffers from a number of problems. First, it is not possible to know the precise identity in anatomical space of the neurons recorded. Also, because the identification of a unit (neuron) depends on the characteristics of the wave form reaching the electrode, subtle shifts in electrode position can create ambiguity in unit identification over long periods of time. This makes studies examining the long-term stability of neural representation or potential anatomical segregation of unit types difficult. Finally, the simultaneous recording of hundreds or thousands of neurons would facilitate the identification of rare cell populations and provide information on the global structure of putative neural representations.

One approach that has been adopted recently to address these issues is the use of imaging, to visually record calcium signals in large populations of neurons. This has been facilitated by the development of genetically encoded fluorescent reporters of intracellular calcium levels. The genetic nature of the indicator allows it to be delivered to specific neural populations by viral or transgenic approaches where it is stably expressed to allow recording over many months. The most widely used reporter (GCAMP) is based on a split version of green fluorescent protein (GFP) linked to a calcium-binding protein [17,18]. The conformational shift in protein structure with calcium binding aligns the GFP moieties to produce fluorescence. The use of direct fluorescent imaging of neural activity allows for precise identification of the neurons recorded and the number of neurons imaged is limited only by the field of view, allowing the activity of 100s to 1000s of neurons to be imaged simultaneously.

GCAMP has been used successfully to image calcium signals in a variety of species from *Caenorhabditis elegans* to mice and rats. The zebrafish is particularly advantageous for this type of imaging study as the animals are largely transparent in early developmental stages. In a recent study, a genetically encoded calcium indicator was used to image activity in most (80%) of the neurons in an awake animal's brain [19]. The study used light-sheet microscopy to rapidly scan large volumes of the brain for fluorescent activation in transgenic animals expressing GCAMP3. Whole brain activity could be scanned every 1.3 s and was examined for correlations in neural activity that would suggest a functionally related circuit. The results showed correlated activity in a hindbrain–spinal cord circuit. The ability to repeatedly image whole brain neural activity would be particularly advantageous for identifying distributed and interacting circuits that underlie specific representations and how they are altered with learning. This would not only provide a better understanding of information flow and processing after learning but also indicate candidate sites for plasticity outside of the typical suspects.

There are number of difficulties associated with similar imaging studies in the mammalian brain. First, the requirement that the animal be tethered to a microscope objective has limited the behavioural repertoire available for study. In addition, limitations on the penetration of light and scattering of signal have generally restricted studies to superficial cortical layers. Two approaches have been used to overcome these limitations to obtain calcium imaging data from the hippocampus in awake, behaving mice. In one study, the animals were head fixed to a microscope objective and allowed to freely run on a ball floating on a column of air [20]. By coupling the animal's movements to visual stimuli in a virtual reality setting, the mice could be allowed to essentially explore a complex spatial environment. In order to clearly image the hippocampus, a column of overlying cortical tissue was removed and the calcium indicator GCAMP3 was delivered to the hippocampus by viral transfection. Using this preparation, the authors were able to image calcium transients in CA1 cell bodies and dendrites from approximately 100 cells simultaneously while the animal navigated through a virtual reality environment. Between 15 and 20% of neurons displayed calcium transients in a location-specific manner. The number of cells with location-specific firing and the size of the firing fields were similar to what is observed using electrophysiological recording and suggests that the calcium indicator is reporting place cell activity. There was no apparent anatomical organization of neurons based on their place fields, a result not accessible with standard recording techniques. This was not unexpected, however it raises the important issue of how to identify and manipulate neurons associated with a specific representation within a given brain area if they are not anatomically segregated.

The second approach that has been developed recently and has the potential to be generally applicable for the imaging of deep brain structures in freely moving animals used an endoscopic microscope [21]. In this case, the machinery for imaging weighs less than 2 g and can be carried on a head mount with the structure to be imaged targeted with stereotactic delivery of the endoscope's fibre optic. This approach was used with GCAMP3 to image calcium signals in hippocampal CA1 neurons over 45 days while the animals ran on a linear track. Between 500 and 1000 cells per mouse were examined over this timeframe to measure the stability of the spatial representation in the hippocampus. As in the previous study, neurons showed clear spatial firing fields consistent with being place cells. In any given session, approximately 20% of neurons met the criteria for place cells; however, the precise ensemble of neurons recruited in each session showed a low level of stability. At five days separation between recording sessions, there was only a 25% overlap in the place cells recorded in any two sessions. The overlap dropped to 15% at 30 days separation between recording sessions, with only 3% of cells active in all 10 recording sessions. So while each day 20% of total hippocampal neurons were active as place cells on the track, the majority of those cells were different from day to day in identical spatial environments. If the hippocampus is representing the environment through the activity of place cells, then why is there not greater stability of the ensemble of neurons activated in the same environment?

There are several possible explanations for the lack of stability in hippocampal ensembles in the preceding experiment. One possibility is that there are subtle differences in the environment from day to day to which the animal is responding. Another possibility is that the task (running for a water reward) was not sufficiently salient to produce a stable representation. A previous study in mice using tetrode recordings showed that the stability of place cells over several days is modulated by the salience of the task that the animals were required to perform in that environment [22]. Having non-food deprived animals collect food pellets during the recording caused the place cells to be unstable from day to day while requiring the animals to navigate to a specific location to avoid an aversive light/noise cue produced the greatest temporal stability in place cell firing. Finally, it is possible that the critical spatial signal is contained in the small percentage of neurons that do show consistent firing between two recording sessions. An examination of the data showed that the information content of the stable component of the ensemble between any two days was more than sufficient to reliably predict the animal's location [21]. Whatever the explanation, this experiment raises an important question in understanding how memories are represented. How consistent is the pattern of brain activity in response to two identical sensory inputs or two different memory recall events? What is noise and what is signal in the pattern of neural activity that is observed? Certainly the brain's ability to consistently recognize and learn about elements in the environment implies some coherent signal in the neural activity patterns induced by the same sensory stimulation, but the models and approaches to understanding this information will differ depending on whether the signal is a dominant or a minor component of the sensory-evoked activity.

## 3. Finding the engram criteria 1 and 2: cellular mechanisms of memory formation

Irrespective of the circuit structure of memory representations in the hippocampus, experiments can be designed to probe the potential mechanisms that underlie information storage more globally in the structure. These experiments have primarily focused on the hypothesis that NMDA-dependent forms of synaptic plasticity, for example LTP, underlie the formation of memory. The first criterion that should be met for any cellular mechanism of memory is that it be induced in the relevant circuit during learning. The greatest difficulty in meeting this requirement is the assumed sparse and distributed nature of the neurons that participate in a given memory and, for a synaptic mechanism such as LTP, the expected limited number of synapses on these cells that would undergo the plasticity. The primary support for learning-induced LTP in the hippocampus comes from a study using inhibitory avoidance learning in rats [23]. Animals were implanted with an array of eight recording electrodes throughout the apical dendritic layer of CA1 and synaptic responses to Schaffer collateral stimulation were recorded before and after training in the avoidance paradigm. Fourteen of 44 recording electrodes from trained animals showed an increase in excitatory postsynaptic potential of 10% or more above baseline, suggesting an anatomically localized learning-induced potentiation of synaptic transmission. To test whether this behaviourally induced potentiation was mechanistically similar to LTP induced by high-frequency stimulation, the authors performed an occlusion experiment. The principle of the occlusion experiment is that as LTP can be saturated, if some fraction of synapses has already undergone LTP, then the total amount of potentiation that can be produced by further stimulation should be reduced. When the amount of potentiation that could be produced by high-frequency Schaffer collateral stimulation at each of the implanted electrodes was examined, it was found that those electrodes that showed the behaviourally induced potentiation had reduced levels of stimulation-induced potentiation. This suggests that the behavioural potentiation occluded LTP induction, and therefore probably used common underlying mechanisms.

Does the behaviourally induced potentiation observed in this study satisfy the first criterion in the search for a hippocampal engram? The answer would seem to be yes, but there are a number of caveats and predictions that arise from this result. First, as the behaviourally induced potentiation was seen on only 30% of the electrodes, this would suggest that the plasticity shows significant anatomical localization. This was not seen in the imaging studies examining neural activity in place cells, that is, neurons encoding similar locations did not show any discernible anatomical clustering [20]. It is possible that in paradigms such as inhibitory avoidance there is a greater functional segregation of neurons encoding relevant aspects of the task and that this is the basis for the localized plasticity effect. It would be interesting to test, for example, whether neurons in the vicinity of the electrodes that showed behaviourally induced potentiation showed greater activation during learning or recall of the task, as might be expected from these results. While these results are intriguing, it will be important to integrate them with more defined circuit analysis and quantification of the specific cells and synapses that are modified.

The most commonly studied form of LTP, and the form seen at hippocampal CA1 synapses, is NMDA receptor dependent, providing an easy experimental manipulation to test the second criterion for a cellular mechanism of memory: does blocking LTP block hippocampal-dependent learning? Early studies using pharmacological inhibition of NMDA receptors showed that bilateral hippocampal infusion of the receptor antagonist AP5 impaired spatial learning in the water maze, suggesting that the criterion was met [24]. However, subsequent pharmacological studies raised a number of questions on the generality of the NMDA receptor requirement in spatial learning. When animals were pretrained in either a spatial or non-spatial version of the water maze, they were insensitive to NMDA receptor blockade, even when the second maze used different cues and occurred in a different room [25,26]. Spatial learning in the second task was still sensitive to hippocampal lesion, showing that circuits through the hippocampus were still required even though NMDA-dependent plasticity was dispensable. This result has been revisited recently with quantitative and anatomically specific NMDA receptor blockade and replicated the basic finding at inhibitor levels sufficient to block dentate gyrus LTP measured *in vivo* [27].

In addition to the pharmacological approach, a series of studies using cell-type specific genetic deletion of NMDA receptors in different hippocampal subregions have attempted to refine our understanding of the role of plasticity in different elements of the tri-synaptic circuit. In one study [28], the NMDA receptor was deleted specifically in the dentate gyrus granule cells of mice, leading to a loss of LTP at perforant path synapses. The animals were examined in a contextual fear-discrimination task in which they were placed in two different chambers over multiple days and received a foot-shock in one of the chambers. Control animals learned to discriminate between the chambers and expressed a fear response specifically to the shocked chamber, while the knockout animals showed fear in both chambers. Although they eventually learned the discrimination task, the results suggest that NMDA-dependent plasticity in the dentate contributes to the ability of animals to pattern discriminate. This is consistent with a previously postulated role for the dentate based on the connectivity properties of the hippocampal circuit [29].

The CA3 neurons have a dense network of recurrent collaterals and it has been hypothesized that this type of circuit structure could readily perform pattern completion with incomplete input information [29]. This idea was examined by deleting NMDA receptors specifically from CA3 neurons in mice [30]. The animals were tested for spatial learning in the water maze task and were indistinguishable from control mice in their acquisition and retrieval in the standard reference memory version. However, when some of the distal visual cues were removed, the NMDA receptor knockout mice showed impaired spatial memory retrieval consistent with a difficulty in pattern completion. Interestingly, the place fields of neurons recorded in area CA1 from the CA3 NMDA receptor knockout animals showed a reduction in robustness (reduced firing rate, increased size of area represented) compared with controls, and this alteration was specific to the partial cue environment.

While the loss of NMDA receptors in CA3 and dentate result in subtle differences in behavioural performance when the task demands are increased, early studies of mice in which the NMDA receptor was deleted specifically in CA1 neurons produced severe deficits in spatial learning and contextual fear conditioning [31,32]. This suggested that plasticity in CA1 was critical to actually storing spatial information whereas plasticity in the other hippocampal areas served a more refined role in recruiting the correct neural ensembles for encoding or recall. However, a recent study revisited the role of NMDA receptors in CA1 neurons and found a much more subtle effect on spatial learning [33]. A new line of mouse was generated that deleted the NMDA receptor in both CA1 and dentate gyrus neurons. Unlike in the previous reports, when examined in the water maze task this new knockout line performed identically to controls. While the animals could develop a normal spatial memory for platform location, they showed a slight deficit only when a competing ambiguous cue was added to the maze.

There are a number of possible explanations for the disparate results between the different CA1 NMDA knockout studies. One likely possibility is that the more severe phenotype found by Tsien *et al*. [32] is due to loss of NDMA receptor in neurons outside of the CA1 region. In the other region-specific knockouts, the behavioural effects are subtle and seem to come into play only when the task is made more difficult by the addition or removal of cues or when a subtle discrimination between similar contexts is required. Each study used different tasks and manipulations and it would be interesting to compare the different knockout lines side by side to determine whether the animals differ from each other or whether the tasks are simply assaying a common parameter associated with discrimination difficulty. Nevertheless, it seems clear that rodents can form spatial memories even in the absence of hippocampal NMDA receptor function. While there are a number of possible interpretations of these results (for example [27]) taken together, it is difficult to argue that NMDA-dependent LTP in the hippocampus meets the criterion of necessity for a cellular mechanism of spatial learning.

The NMDA receptor and the various forms of synaptic plasticity that it supports display many features that recommend them as a cellular memory mechanism, as had been discussed previously [34]. The results discussed above could simply mean that NMDA-dependent LTP is not used in the hippocampus, in rodents, for the tasks investigated. The hippocampus could still hold complex representations associated with declarative memory but with the critical sites of plasticity located in some upstream processing region. Alternately, there are other forms of synaptic and cellular plasticity that are independent of NMDA receptor function that could support hippocampal circuit modification with memory [35–39]. Finally, it is possible that NMDA-dependent forms of plasticity in the hippocampus do in fact contribute to the formation of memory, but because of the distributed nature of memory representations, eliminating this component of the trace is insufficient to severely disrupt behavioural recall. As this brief discussion illustrates, it is not an easy task to pick the right neurons, the right behaviour and the right molecular mechanism to provide a definitive test of any particular cellular memory mechanism.

## 4. Finding the engram criteria 3 and 4: circuit-based approaches to memory

Criteria 3 and 4 for identifying a complex engram are the most difficult to satisfy as they require identification and manipulation of the neurons and synapses used to represent a given memory, and this will vary for each individual animal and memory task. One useful approach that was developed recently allows the linking of natural patterns of sensory-evoked neural activity to genetic alteration, such that the pattern of neurons activated during a behavioural session can be specifically altered to express essentially any desired protein [40]. This approach offers the possibility of obtaining molecular and electrical control over the precise pattern of neurons recruited in an individual animal during any type and stage of behavioural training.

The immediate early genes (IEGs) are a group of genes that are rapidly and transiently induced by neural activity [41]. They are induced within minutes of a spike burst and turn over rapidly at both the protein and mRNA level, returning to baseline expression within several hours [42]. Staining brain sections for IEG expression thus provides a snap shot of the brain's activity pattern during a 1–2 h time window prior to the sacrifice of the animal. This approach has been used extensively in rodents to map activation in many brain regions and behavioural paradigms, providing a report of neural activity that is generally consistent with physiological studies. For example, one study took advantage of the time delay in the nuclear–cytoplasmic transport of the mRNA for the IEG *arc* to allow the determination of neural activity at two closely spaced time points [43]. They found that when animals were allowed to explore two environments, approximately 30% of neurons in the hippocampus showed *arc* signal for each time point, consistent with the fraction of cells that display place fields in recording studies. The number of cells that were active when animals were exposed twice to the same environment was much higher than when animals explored to two distinct environments. Thus, *arc* gene expression reports a stable environment-specific (spatial) map, at least at short time points, consistent with electrophysiological recordings of place cells.

**Figure 1.**
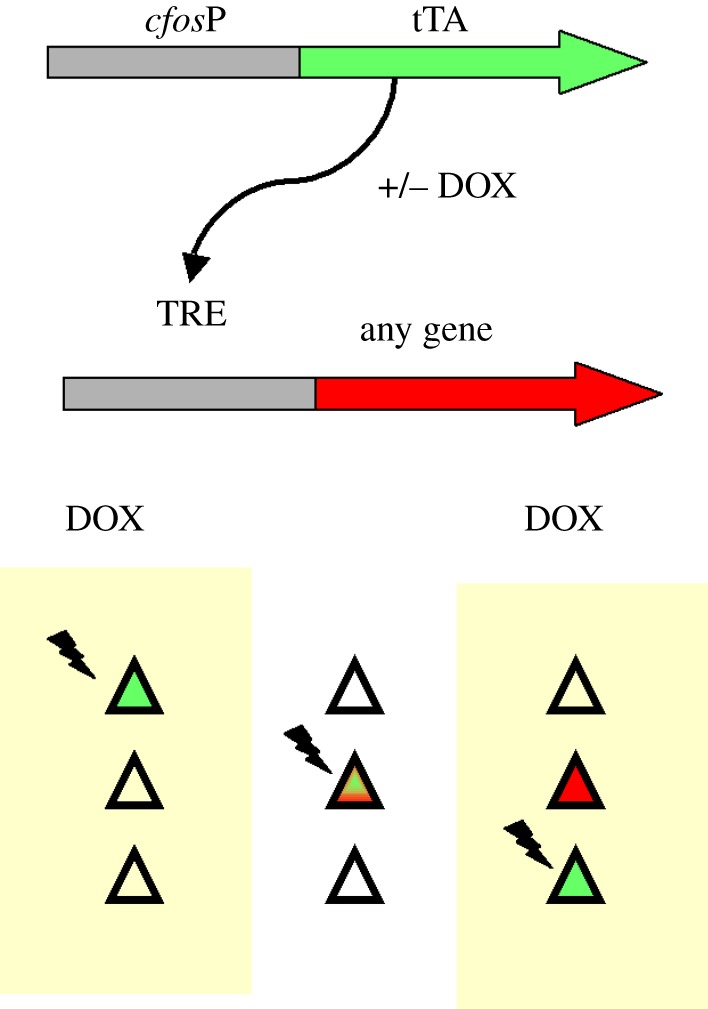
Genetic tagging of active neural ensembles. Mice carry the two transgenes shown. The first transgene links the doxycycline (DOX)-regulated transcription factor tetracycline transactivator (tTA) to the neural activity-dependent *cfos* promoter (*cfos*P). The second transgene links any effector to the tTA-dependent promoter tetracycline responsive element (TRE). Only during a time window when DOX is absent does neural activity drive expression of the TRE-linked effector gene.

The ability of neural activity to induce gene expression allowed the development of a transgenic mouse in which the neurons active during a given time window could be specifically labelled with any gene, as outlined in figure 1 [40]. The mouse carries two separate transgenes. In the first transgene, the promoter for the IEG *cfos* is used to drive expression of the tetracycline transactivator (tTA). This provides neural activity-dependent regulation of the doxycycline (DOX)-dependent transcription factor tTA. The second transgene uses the tetracycline responsive element (TRE) promoter to drive any gene of interest. With this genetic arrangement, as long as the animals are maintained on DOX, the *cfos* promoter will continually drive expression of tTA in active neurons but the TRE-linked transgene will not be expressed. In order to genetically tag active neural ensembles, the DOX is removed from the animal's diet to open a window of time when the *cfos*-driven tTA is functional and allows transcription of the TRE-linked gene in neurons that are electrically active during this DOX-free period. The authors used expression of a TRE-linked marker protein to show that neural ensembles activated during learning in a fear-conditioning task were reactivated in the amygdala during memory retrieval.

The ability to genetically alter neurons based on natural activity patterns induced by environmental stimuli opens up the possibility for a variety of studies focused on probing the functional relevance of these ensembles. This approach was used to test the nature of the neural representation for a hippocampal-dependent memory [44]. By using the *cfos*-tTA transgenic mouse discussed above in conjunction with a TRE-driven channelrhodopsin2 (ChR2) delivered on a viral vector, the authors were able to express ChR2 specifically in neurons that were activated during learning in a contextual fear-conditioning task. Animals received foot-shocks in the training context (BoxA) in the absence of Dox, to allow the expression of ChR2 in neurons that were sufficiently active to express the *cfos*-tTA transgene. This produced a standard fear memory of BoxA. When the animals were placed in a neutral context (BoxB) they did not show a fear response, however when light pulses were delivered to the dentate gyrus to stimulate the ChR2-expressing neurons, the animals showed fear. When the set of neurons activated in the third neutral context (BoxC) were labelled with ChR2, their stimulation did not induce a fear response, showing specificity of the ensemble. This suggests that artificial stimulation of the dentate gyrus neurons active during learning in BoxA recruited a component of the fear memory representation essentially causing the animals to ‘think’ they were in the conditioning BoxA.

This is a critical finding as it links, for the first time, a specific pattern of neural activity in the brain to a complex external stimulus. But is this an engram for conditioned fear? Based on the criteria outlined in the introduction, the answer would be no. In contextual fear conditioning, the animal is forming an association between a specific environment (the Box) and an aversive event (the shock). The neurons of the engram would be the locus of molecular and cellular change that allow this association; however, there is no indication that this is the case in these experiments. Given what we know about the hippocampus, the most likely interpretation is that the stimulation of the dentate gyrus neurons produces a representation of the context and as this context was associated with shock, its activation will produce a fear response. Where the plasticity occurred to produce that link is not clear. To illustrate this point, assume that we could artificially stimulate the retina of a fear-conditioned animal in the same manner as when the animal naturally explored the conditioning chamber in which it received a shock. This would likely cause the animal to perceive that it was in the conditioning chamber, and thereby produce the fear response, but it would not mean that the engram for that fear memory was contained in the retina. Nevertheless, this study is a required first step in the search for the engram as it provides important information on the nature of complex representations in the hippocampus and illustrates techniques to probe their structure and function.

An alternative to light-gated channel control of neural activity by optogenetics is a chemical genetic approach using designer receptors or DREADDs. One such designer receptor (hM3Dq) is a Gq-coupled human muscarinic receptor that has been mutated to no longer respond to acetylcholine but instead responds to the synthetic ligand clozapine-N-oxide (CNO) [45]. In hippocampal pyramidal cells, activation of hM3Dq by CNO results in a 5–8 mV depolarization and subsequent increase in action potential firing. Garner *et al*. [46] used the *cfos*-based genetic tagging approach to control the activity of specific neural ensembles with hM3Dq to probe the role of internally generated neural representations during contextual fear conditioning. The authors tagged the ensemble of neurons activated in one context (BoxA) with the hM3Dq receptor, and then stimulated those neurons with CNO while delivering foot-shocks in a separate context (BoxB). The expression of the receptor (expressed in BoxA responsive neurons) was widely distributed throughout the hippocampus and cortex and, not surprisingly, depolarization of these neurons during conditioning interfered with the animal's ability to form a fear memory for the conditioning context (BoxB). The animals also failed to develop a fear response to BoxA or to the artificial stimulation of the BoxA-tagged neurons by CNO, suggesting that this form of electrical manipulation was not sufficient to recruit a functional representation of BoxA as was seen with the more local light-based stimulation in the dentate gyrus [44]. However, when animals were placed in the conditioning chamber in which they were shocked (BoxB) while the BoxA neurons were stimulated with CNO, they showed a fear response that approached that of control animals. This suggested that the animals had formed a hybrid neural representation incorporating elements of the natural sensory activity from BoxB with the artificially generated activity of the CNO-stimulated BoxA neurons.

Does this experiment, with highly artificial modes of neural activation, tell us anything about learning mechanisms under natural conditions? One point that is often lost sight of in a typical study is that the brain is not a blank slate at the start of the experiment, nor is the brain silent in the absence of experimenter-provided stimuli or responding exclusively to those stimuli during the experiment. There is extensive internally generated ‘spontaneous’ activity in addition to activity evoked by the experimental cues. What is this spontaneous activity, is it just noise or does it have a function? Recordings of place cells in the hippocampus have shown that following a typical session in which animals explore a distinct environment, the ‘spontaneous’ off-line activity tends to display a temporal structure that parallels that seen during the actual exploration [47–49]. That is, there is a kind of off-line replay of the neural ensembles associated with the actual experience. Similar off-line replay of sensory-evoked activity has been described in other brain areas such as visual cortex [48,50]. So it seems that while internally generated activity is spontaneous, it is not random, but is made up of ensembles that represent elements of previous experience. In this study [46], the neurons associated with the previous experience of exploring BoxA were specifically activated while the animal was learning an aversive association in BoxB and in order to produce fear recall the conjunctive activation of BoxA neurons was also required. A similar process must be common in complex forms of learning where new information is integrated with old information to form complex knowledge schemas. For example, in learning a new mathematical concept it must be integrated with previous information that is represented by internally generated activity. The cue of walking into the maths class or exposure to a new concept is likely to activate internal representations (that may be conscious or unconscious) related to the schema for that subject. This internally generated activity, representing previously learned mathematical information, will facilitate the integration of the new sensory-driven information, i.e. the new concepts being taught in class. In the previous mouse experiment, this mechanism was demonstrated, albeit in a somewhat crude and highly artificial manner. The distributed representation of a past experience (BoxA) was forced to become active during learning new information (BoxB) and it was demonstrated that two representations were integrated.

While these experiments illustrate some interesting points about how the brain can use distributed ensembles of neurons to represent elements of the environment, they do not get us closer to identifying the key sites of plasticity that underlie memory encoding. Nevertheless, they provide a technical approach and proof of principle for probing nervous system function at the level of specific circuits. In essence, they address the problem of studying a process for which the individual cells involved will vary from animal to animal and task to task. The genetic tagging of active circuits in conjunction with new approaches for examining patterns of neural activity over large brain areas with cellular resolution provide a framework for designing experiments to address the four criteria for identifying and understanding the function of an engram for complex forms of declarative memory.

## 5. Summary and framework

Much of what we know about the processing of information in the brain is derived from measures of sensory-evoked neural responses. These studies often examine a limited number of neurons in a small region of the brain over a short time period. We know that memories can be maintained for extremely long times and are presumably represented in some component of neural activity. A key question that must be addressed before we can begin the search for an engram is to determine the extent of signal to noise in the patterns of activity associated with memory. If we could map the activity of every neuron in the brain during exposure to two identical complex sensory inputs how similar would the two patterns be? Would they be closer to 100% or 0%? The technology is now available to address this question in zebrafish and in a more limited way in mammalian models using calcium imaging, and it seems that the answer may be closer to 0% than 100%, depending on brain area and time between experiences. In primary somatosensory and piriform (olfactory) cortices, ensemble responses to repeated sensory stimuli show approximately 70% concordance over trials [51,52]. This would seem to provide an upper limit to signal strength for a simple sensory representation in the cortex. By contrast, in the hippocampus, similar imaging studies found only 15–25% of place cells were active in any two sessions exploring the same environment and only 3% were active over all sessions in a 45-day period [21]. This suggests either that there are multiple hippocampal spatial representations for any given environment or that the stable spatial map involves only a very limited number of neurons. Understanding the nature of complex neural representations is critical as it will restrict the possible forms that an engram can take and the techniques to employ in defining the sites of plasticity.

The ability to image neural activity with anatomically precise single-cell resolution provides a means to search for the sparse population of neurons that might undergo the critical plasticity of the engram even with the complex stimuli of a declarative memory. For example, in contextual fear conditioning, neurons that were responsive to context alone or to shock alone could be recorded and neurons that shifted activity (newly active or silenced) during the conditioning or memory retrieval phase would represent excellent candidates as sites of learning-based plasticity. This could be tested by directed recording from these identified populations to test for changes in excitability, synaptic responses or morphology. Examination of a large number of areas, up to the entire brain, would be useful to expand the search beyond existing models of where to look. The zebrafish is particularly well suited for whole brain activity mapping, and one recent study used calcium imaging to identify a region of the brain that was specifically activated by conditioning in an avoidance task [53]. In the mouse, the combination of IEG activity-based labelling and new high-throughput whole brain imaging techniques could allow this type of study, albeit with the limited temporal resolution of IEG reporters [54].

**Figure 2.**
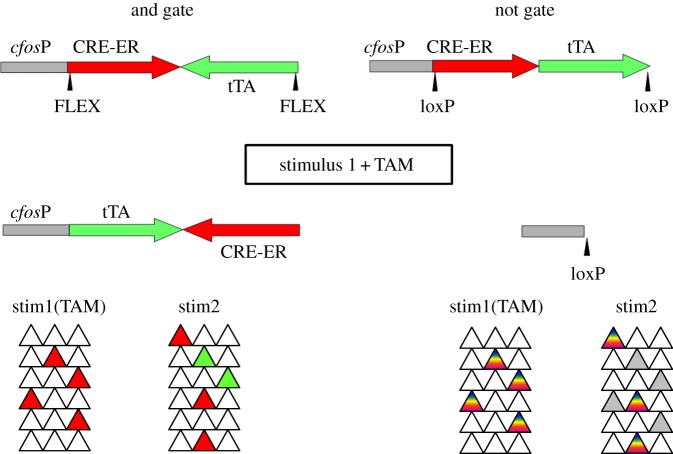
Contingent neural activity-based genetic switches. Genetic arrangement to produce ‘and’ gated (requires activity at two time points) and ‘not’ gated (requires activity at the second, not first, time point). The readout of the gates is functional expression of tTA from the *cfos* promoter (*cfos*-P) and activation of a TRE-linked transgene. Bottom panel shows gene expression in neural ensembles based on activity to two stimuli. For the ‘and’ gate, the tTA-expressing neurons (green) were active with both stimuli because tTA can only be expressed following the induced recombination in cells active during stimulus 1. For the ‘not’ gate, the neurons activated by stimulus 1 (red + green) undergo recombination to remove the CRE/tTA transgene which then cannot be expressed in response to stimulus 2 (grey) FLEX, elements mediating DNA inversion in response to CRE recombinase. loxP, elements mediating DNA deletion in response to CRE recombinase. CRE-ER, tamoxifen inducible CRE recombinase. TAM, tamoxifen.

New imaging techniques can help to define the structure of neural representations and identify neural populations that may undergo the cellular modifications with learning that form the basis of the engram. However, to test the role of these cellular modifications and to understand how they function within the brain to modify information processing will require direct manipulation. The use of IEG promoters in combination with regulated genetic switches provides a means of molecularly altering specific neural ensembles based on their sensory-evoked activity. However, the neurons of the engram are undoubtedly a much more limited population than simply active neurons, and for a more precise capacity for manipulation it would be useful to have the ability to genetically alter neurons based on different firing contingences, for example, tagging only neurons that were active during pairing of a conditioned (CS) and unconditioned stimulus (US) but not either CS or US alone. Figure 2 shows two genetic arrangements that would allow molecular tagging of active neural populations using a contingent ‘and’ gate (only populations active at both of two time points) and a ‘not’ gate (only neurons active at one time point but not another). The strategy combines both the CRE/loxP system and the TET system in conjunction with the *cfos* promoter. For the ‘and’ gate, a tamoxifen inducible CRE-ERT2 and an inverted tTA are linked in tandem to the *cfos* promoter and flanked by FLEX elements, which are designed to invert the bounded DNA in response to CRE-recombinase [55]. During the first sensory stimulation, the animal receives tamoxifen to activate CRE-recombinase causing an inversion event that now links tTA expression directly to the *cfos* promoter, providing a genetic arrangement identical to that shown in figure 1. During the second stimulus, DOX is removed allowing tTA to drive expression of any TRE-linked transgene. In this configuration, the only neurons that will express the TRE-linked transgene are those that are sufficiently active to express *cfos* during both sensory stimuli. In the ‘not’ gated system, both the inducible CRE and tTA are functionally expressed from the *cfos* promoter and the entire cassette is flanked by loxP sites. The drug treatments and behavioural manipulations are identical to the ‘and’ gate, except that in this case the active neurons that express CRE during the first stimulus will delete the entire cassette preventing any future activation of a TRE-linked transgene in that population of cells. Therefore, the only neurons that will be labelled are those that were active during the second stimulus but not during the first stimulus. With such systems, very precise populations of neurons could be modified with any genetically encoded marker or effector to control activity, signalling pathways, gene expression or to label fibres for anatomical studies.

The past 5 years in neuroscience has seen an expansion of new techniques for examining neural activity in large populations of neurons with greater anatomical specificity. In addition, tools such as optogenetic and DREADD proteins allow the direct manipulation of the activity and biochemistry of genetically defined populations of neurons. In combination with IEG-driven genetic modification, these tools can be used to probe the function of widely dispersed neural ensembles to begin to directly test neural coding properties. Future improvements such as increasing the temporal resolution, sensitivity, anatomical breadth and computational analysis capabilities of activity measures will facilitate the search for specific populations of neurons that are altered with learning. Parallel improvements in the temporal resolution and contingency-based genetic manipulation will allow molecular control of neural populations based on subtle features of their natural response properties. Together, these approaches provide a technical framework for identifying key properties of the engram for complex forms of hippocampal-dependent declarative memory.
